# Age‐related impact of outcomes in hospitalized patients with alcohol overuse

**DOI:** 10.1111/acer.70219

**Published:** 2025-12-01

**Authors:** Dhweeja Dasarathy, Amy H. Attaway

**Affiliations:** ^1^ Department of Internal Medicine Stanford University Stanford California USA; ^2^ Department of Pulmonary Cleveland Clinic Cleveland Ohio USA

**Keywords:** aging population, alcohol use disorder, frailty, healthcare utilization, sarcopenia

## Abstract

**Background:**

The incidence of alcohol use disorder (AUD) and related complications, such as alcohol‐associated liver disease and alcohol‐associated cirrhosis, continues to rise in the United States. However, limited data exist on the impact of AUD in aging hospitalized populations. We aimed to evaluate the association between AUD and clinical outcomes in aging hospitalized patients.

**Methods:**

We analyzed 2017–2020 data from the Nationwide Inpatient Sample, including adults aged 18–80 with and without AUD based on ICD‐10 codes (AUD: *n* = 523,464; general medical population [GMP]: *n* = 238,678). Primary outcomes included length of stay, discharge disposition, healthcare cost, mortality, sarcopenia, and frailty. Multivariable regression analyses were conducted within and across age strata (<50, 50–60, 61–70, 71–80 years).

**Results:**

Compared to the GMP, patients with AUD had higher healthcare utilization and more comorbidities across all age groups. A higher proportion of females were diagnosed with AUD in each age category. The prevalence of sarcopenia and frailty increased with age, with the highest rates in AUD patients over 70. Across all age groups, AUD was independently associated with worse outcomes, including increased sarcopenia, frailty, healthcare costs, longer hospital stays, and worse discharge dispositions. Mortality risk was elevated in AUD patients under 70 (odds ratios: 1.55, 1.41, 1.21 for <50, 50–60, 61–70, respectively), but not in those over 70 (OR: 0.95), suggesting survivor bias. Concurrent sarcopenia and frailty conferred greater mortality risk than either alone.

**Conclusions:**

AUD is independently associated with worse clinical outcomes in hospitalized adults, especially older adults. These findings underscore the urgent need for age‐tailored alcohol intervention strategies.

## Graphical abstract

In the Nationwide Inpatient Sample, alcohol use disorder (AUD) was independently associated with increased frailty, sarcopenia, longer hospital stays, higher costs, and adverse discharge outcomes across all age groups. Mortality was increased in AUD patients under 70 but not in those older than 70, suggesting survivor bias. The coexistence of frailty and sarcopenia conferred the highest mortality risk. Our findings highlight the need for targeted alcohol interventions and frailty/sarcopenia screening in older hospitalized patients with AUD.

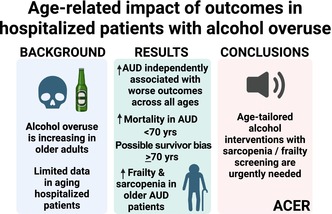

## INTRODUCTION

The prevalence and incidence of alcohol use and alcohol‐related tissue injury continue to increase in the United States (Turner & Mathias, 2018). Alcohol use disorder (AUD) affects 28.9 million people over the age of 12 and has a significant annual economic burden of approximately $249 billion according to National Institute on Alcohol Abuse and Alcoholism (2024). Even though the best studied consequences of alcohol include alcohol overuse and alcohol‐related liver disease, the incidence and consequences of alcohol overuse especially in the aging population are unknown. There are emerging data that alcohol use among the elderly is increasing, with studies finding that 10.7% of adults aged 65 and older engaged in alcohol binging within the past month and 2.3% of adults aged 65 years and older meet criteria for alcohol use disorder within the past 12 months (Joshi et al., 2021). Furthermore, between 2002 and 2019, the percentage of people who reported drinking at least one alcoholic beverage per month increased by 16% (National Institute on Alcohol Abuse and Alcoholism, 2024). Given the multiple comorbidities that can develop with increasing age, the clinical consequences and utilization of healthcare resources are likely to be higher in the elderly with chronic alcohol use. Limited studies have found that older adults who consume alcohol beyond the recommended guidelines are at increased risk of liver disease, diabetes, congestive heart failure, depression, and anxiety (Barry & Blow, 2016). Despite the clinical significance, increased prevalence of age‐related alcohol use disorder, and increased health risks associated with age‐related alcohol use, there are limited data on the age‐related impact of outcomes of hospitalized patients with alcohol overuse.

In our study, we used the Nationwide Inpatient Sample (NIS) database to investigate the impact of alcohol overuse across the lifespan in terms of clinical outcomes, as compared to a control population of aged medical patients without a diagnosis of alcohol overuse. Our analyses showed that alcohol use disorder (AUD) is independently associated with worse clinical outcomes in hospitalized adults, especially older adults. Our analyses highlight the need for age‐tailored alcohol intervention strategies in patients with AUD.

## METHODS

The Nationwide Inpatient Sample (NIS) is a large cross‐sectional database developed by the Healthcare Cost and Utilization Project (HCUP) Agency for Healthcare Research and Quality. The NIS comprises administrative data from over 8 million hospitalizations per year including 1000 United States community hospitals and represents approximately 20% of all hospitalizations in the United States. In the present study, adult patients (≥18 years to 80 years old) were included with AUD defined using *International Statistical Classification of Diseases and Related Health Problems, Tenth Revision* (*ICD‐10*) discharge diagnosis codes for alcohol abuse, dependence, and use (F10.0, F10.1, F10.2, F10.9x), excluding “in remission” specifiers (F10.11, F10.21, F10.91) (Bernstein et al., 2024; Kim et al., 2012). Author A.H.A. completed the requisite HCUP data use agreement and coursework. Because the NIS represents deidentified data that are publicly available, our institution does not require IRB (Institutional Review Board) approval. We analyzed the data from January 1, 2017 to December 31, 2020.

The primary and secondary diagnoses associated with the ICD codes for alcohol use disorder were analyzed. We included a comparison group of a concurrent, random sample (2%) of general medical patients (GMP) within the NIS without alcohol use disorder. Clinical outcomes included length of stay (LOS), discharge disposition, healthcare cost, and mortality. Covariates with counts less than or equal to 10 were excluded based on the NIS privacy agreement. As the dataset in the NIS is de‐identified and does not incorporate readmission status, readmission of the same patient to the hospital in the same year is considered a separate encounter.

Summary statistics included counts and percentages for categorical variables and means with standard deviation for continuous variables that were normally distributed. When comparing between groups, we used a *z*‐test of proportions for categorical data, a *t*‐test for normally distributed continuous data, and a Mann–Whitney test for non‐parametric data. Box–Cox analysis was performed for data that were not normally distributed including hospital cost and LOS, which were then log‐transformed. Multivariate logistic regression analysis was performed to identify independent associations with categorical clinical outcomes including mortality and discharge status. Log‐transformed multivariate linear regression analyses were performed to identify independent associations with hospital cost and LOS, and beta coefficients were then exponentiated. Models were constructed using variables known to impact clinical outcomes related to age‐related alcohol use including sex and race, and comorbidities were incorporated using the Elixhauser comorbidity score. A sub‐analysis was performed analyzing those with alcohol use disorder and concomitant alcohol‐associated liver disease (ICD10 K70.0, K70.1, K70.2, and K70.9), alcohol‐associated cirrhosis (ICD10 codes K70.3), and alpha 1 antitrypsin deficiency (ICD10 E88.01). Social determinants of health risk factors (SDOHr) were defined by ICD‐10 Z‐codes flagged in the chart related to housing and economic status, unemployment, occupation, psychosocial, family, and healthcare access (ICD10 Z55‐57, Z59, Z60, Z62‐65, Z75) (Luke & Scribano, 2023). The ICD10 code definitions are summarized in Table S1. For comparisons across multiple age categories in the same group, we used ANOVA with post hoc Tukey HSD for multiple comparisons. All analyses were two‐tailed and performed at a significance level of 0.05. Confidence intervals (CI), unless otherwise stated, were 95%. R version 4.0.2. (The R Foundation for Statistical Computing, Vienna, Austria).

## RESULTS

Demographic characteristics of the overall patient population including patients with (AUD) and without alcohol use disorder (general medical population or GMP) are shown in Table 1. Patients with (*n* = 523,464) and without AUD (*n* = 238,678) were categorized by age (<50, 50–60, 61–70, and 71–80). Statistically significant differences in gender were noted across age categories for the GMP and AUD, with males contributing to a greater proportion of patients with AUD compared to GMP. There were also significantly greater proportions of those with a social determinant of health risk factor (SDOHr) among the patients with AUD versus the GMP (*p* < 0.001). For all age categories, AUD had a greater association with adverse clinical outcomes, including sarcopenia, frailty, healthcare cost, and in‐hospital mortality, and adverse discharge status. Sarcopenia + frailty was also higher across all age groups in the AUD group compared to the GMP (*p* < 0.001). Statistically significant differences were noted between GMP and AUD when comparing comorbidities across age categories. Congestive heart failure, diabetes mellitus type 2, anemia, and chronic kidney disease were noted to be less in AUD patients compared to GMP across age groups, whereas COPD and alpha 1 anti‐trypsin deficiency were greater in AUD patients compared to GMP across age groups. Furthermore, post hoc analysis of ANOVA results revealed statistically significant differences among all age groups within either GMP or AUD groups for age, gender, race, insurance, and comorbidities.

**TABLE 1:** Demographic and clinical characteristics of hospitalized patients with and without alcohol use disorder.

Age categories in years	Patients without AUD (*n* = 238,678)					Patients with AUD (523,464)				
	<50 (*n* = 75,435)	50–60 (*n* = 46,838)	61–70 (*n* = 57,190)	71–80 (*n* = 59,215)	*p* Value	<50 (*n* = 199,711)	50–60 (*n* = 155,388)	61–70 (*n* = 119,131)	71–80 (*n* = 49,234)	*p* Value
Age (mean (SD))	36.26 (9.04)	55.79 (2.81)	65.49 (2.86)	75.72 (3.13)	<0.001*	38.43 (8.28)A	55.68 (2.78)B	64.84 (2.79)C	74.80 (2.99)D	<0.001*
Female (%)	39,708 (52.6)	21,168 (45.2)	27,180 (47.5)	31,029 (52.4)	<0.001*	63,618 (31.9)A	41,855 (26.9)B	28,929 (24.3)C	12,909 (26.2)D	<0.001*
Race (%)										
White	38,237 (51.8)	28,025 (61.1)	37,814 (67.4)	43,128 (74.2)	<0.001*	112,149 (57.5)A	95,484 (62.6)B	79,383 (67.8)c	37,186 (76.8)D	<0.001*
Black	16,842 (22.8)	9751 (21.2)	9735 (17.4)	6839 (11.8)	<0.001*	40,468 (20.8)A	35,180 (23.1)C	23,887 (20.4)C	6205 (12.8)D	<0.001
Other	18,763 (25.4)	8114 (17.7)	8519 (15.2)	8142 (14.0)	<0.001*	42,390 (21.7)A	21,895 (14.4)B	13,729 (11.7)C	5009 (10.3)D	<0.001*
Mean household income by zip code (%)										
0–25th percentile	26,126 (35.5)	16,234 (35.6)	18,136 (32.3)	16,255 (27.9)	<0.001	69,331 (36.1)a	58,529 (39.2)B	41,972 (36.4)C	13,836 (28.7)d	<0.001*
26–50th percentile	19,437 (26.4)	11,944 (26.2)	14,945 (26.6)	15,505 (26.6)	<0.001	50,371 (26.2)	38,733 (25.9)	30,060 (26.1)c	12,525 (26.0)d	<0.001
51st to 75th percentile	15,998 (21.7)	9990 (21.9)	13,090 (23.3)	14,342 (24.6)	<0.001	42,634 (22.2)a	30,961 (20.7)B	25,301 (21.9)C	11,684 (24.2)d	<0.001
76–100th percentile	12,046 (16.4)	7462 (16.4)	9933 (17.7)	12,209 (20.9)	<0.001	29,785 (15.5)A	21,075 (14.1)B	17,957 (15.6)C	10,141 (21.0)	<0.001
Urban–rural classification for US county (%)										
Central counties of metro areas of ≥1 million	26,513 (35.6)	15,099 (32.6)	17,275 (30.4)	16,407 (27.8)	<0.001*	68,550 (35.2)	52,303 (34.5)B	38,401 (32.8)C	14,941 (30.6)D	<0.001*
Fringe counties of metro areas of ≥1 million	17,388 (23.4)	11,046 (23.9)	13,600 (23.9)	15,155 (25.7)	<0.001	44,111 (22.7)A	33,655 (22.2)B	26,201 (22.4)C	12,086 (24.7)D	<0.001
Counties in metro areas of 250,000–999,999	15,672 (21.1)	9880 (21.4)	12,311 (21.7)	12,875 (21.8)	<0.001	42,698 (21.9)A	33,779 (22.3)B	26,209 (22.4)C	10,954 (22.4)d	<0.001
Counties in metro areas of 50,000–249,999	6467 (8.7)	4227 (9.1)	5529 (9.7)	5987 (10.1)	<0.001*	17,884 (9.2)A	14,030 (9.3)	11,468 (9.8)	4878 (10.0)	<0.001
Micropolitan counties	5130 (6.9)	3607 (7.8)	4817 (8.5)	5195 (8.8)	<0.001*	13,400 (6.9)	10,927 (7.2)B	9137 (7.8)C	3704 (7.6)D	<0.001
Not metropolitan or micropolitan counties	3248 (4.4)	2402 (5.2)	3301 (5.8)	3441 (5.8)	<0.001	7907 (4.1)A	6713 (4.4)B	5571 (4.8)C	2322 (4.7)D	<0.001
Primary payer for insurance (%)										
Medicare	8994 (11.9)	12,649 (27.0)	35,937 (62.9)	53,104 (89.8)	<0.001*	18,165 (9.1)A	31,202 (20.1)B	66,256 (55.7)C	43,925 (89.3)d	<0.001*
Medicaid	28,974 (38.5)	12,841 (27.5)	5807 (10.2)	979 (1.7)	<0.001*	86,713 (43.5)A	60,211 (38.8)B	20,095 (16.9)C	590 (1.2)D	<0.001*
Private insurance	24,833 (33.0)	15,722 (33.6)	12,149 (21.3)	3713 (6.3)	<0.001*	48,340 (24.3)A	38,320 (24.7)B	21,847 (18.4)C	3078 (6.3)D	<0.001*
Other	12,497 (16.6)	5557 (11.9)	3233 (5.7)	1368 (2.3)	<0.001*	46,087 (23.1)A	25,413 (16.4)B	10,753 (9.0)C	1603 (3.3)D	<0.001*
Comorbidities (%)										
CHF	5048 (6.7)	8475 (18.1)	14,186 (24.8)	18,433 (31.1)	<0.001*	12,059 (6.0)A	23,155 (14.9)B	25,451 (21.4)C	13,175 (26.8)D	<0.001*
COPD	3331 (4.4)	9858 (21.0)	15,653 (27.4)	16,760 (28.3)	<0.001*	10,739 (5.4)A	37,376 (24.1)B	40,066 (33.6)C	17,418 (35.4)D	<0.001*
Alpha 1 antitrypsin deficiency	847 (1.1)	796 (1.7)	942 (1.6)	793 (1.3)	<0.001	4063 (2.0)A	3995 (2.6)B	2745 (2.3)C	930 (1.9)D	<0.001*
CKD	5983 (7.9)	8143 (17.4)	13,676 (23.9)	17,993 (30.4)	<0.001*	8432 (4.2)A	14,957 (9.6)B	16,562 (13.9)C	10,040 (20.4)D	<0.001*
Type II DM	10,912 (14.5)	16,202 (34.6)	23,220 (40.6)	24,334 (41.1)	<0.001*	22,942 (11.5)A	32,903 (21.2)B	27,115 (22.8)C	11,612 (23.6)D	<0.001*
Anemia	3739 (5.0)	3949 (8.4)	5663 (9.9)	6012 (10.2)	<0.001*	6169 (3.1)	8517 (5.5)	7576 (6.4)	3496 (7.1)	<0.001*
Elixhauser comorbidity score	21.86 (11.80)	25.60 (12.26)	27.25 (12.48)	28.28 (12.48)	<0.001*	19.49 (12.00)A	22.47 (12.38)B	23.59 (12.43)C	24.36 (12.47)D	<0.001*
Clinical outcomes										
Disposition of patient upon discharge (%)										
Routine discharge	60,703 (81.4)	32,071 (70.0)	32,704 (59.0)	26,470 (46.7)	<0.001*	157,263 (80.0)A	108,121 (71.6)B	67,764 (58.9)	20,656 (43.7)D	<0.001*
Transfer to short‐term hospital	1518 (2.0)	1221 (2.7)	1734 (3.1)	1519 (2.7)	<0.001	4191 (2.1)	3801 (2.5)b	3295 (2.9)c	1221 (2.6)	<0.001
Transfer to other: SNF, ICF	4365 (5.9)	5360 (11.7)	10,547 (19.0)	16,044 (28.3)	<0.001*	14,418 (7.3)A	17,815 (11.8)	23,850 (20.7)C	15,181 (32.1)D	<0.001*
Transfer to Home Health Care (HHC)	4332 (5.8)	5689 (12.4)	9545 (17.2)	12,268 (21.6)	<0.001*	7102 (3.6)A	13,713 (9.1)B	16,287 (14.2)C	9461 (20.0)D	<0.001*
Against medical advice (AMA)	3682 (4.9)	1445 (3.2)	875 (1.6)	417 (0.7)	<0.001*	13,582 (6.9)A	7549 (5.0)B	3824 (3.3)C	790 (1.7)D	<0.001*
Total charges in $ (median (IQR))	28,355 [16,195, 52,744]	35,571 [20,015, 67,508]	38233 [21409, 717845]	38146 [21570, 70930]	<0.001	26,677 [15,189, 50,080]A	33,784 [19,126, 63,121]B	38,770 [22,014, 72,475]C	41,609 [23,452, 76,477]D	<0.001*
Length of stay in days (median (IQR))	3.0 [2.0, 5.0]	3.0 [2.0, 6.0]	4.0 [2.0, 6.0]	4.0 [2.0, 6.0]	<0.001	3.0 [2.0, 5.0]	3.0 [2.0, 6.0]	4.0 [2.0, 7.0]C	4.0 [2.0, 7.0]D	<0.001
In‐hospital mortality (%)	784 (1.0)	1027 (2.2)	1765 (3.1)	2472 (4.2)	<0.001*	2957 (1.5)A	4309 (2.8)B	4036 (3.4)C	1900 (3.9)d	<0.001*
Number of ICD‐10‐CM on discharge (mean (SD))	10.84 (6.29)	14.53 (6.66)	16.06 (6.67)	17.09 (6.50)	<0.001*	12.64 (5.89)A	15.40 (6.25)B	16.92 (6.34)C	18.14 (6.28)D	<0.001*
SDOHr (%)	4484 (5.9)	2087 (4.5)	1200 (2.1)	590 (1.0)	<0.001*	25,826 (12.9)a	15,821 (10.2)b	7694 (6.5)c	1423 (2.9)d	<0.001*
Sarcopenia (%)	3464 (4.6)	3814 (8.1)	5676 (9.9)	6710 (11.3)	<0.001*	12,581 (6.3)A	17,491 (11.3)B	17,895 (15.0)C	8083 (16.4)D	<0.001*
Frailty (%)										
Low risk	53,919 (71.5)	25,652 (54.8)	26,109 (45.7)	21,447 (36.2)	<0.001*	130,127 (65.2)A	78,134 (50.3)B	46,415 (39.0)C	13,927 (28.3)D	<0.001*
Intermediate risk	20,736 (27.5)	19,872 (42.4)	28,493 (49.8)	33,445 (56.5)	<0.001*	67,270 (33.7)A	72,805 (46.9)B	66,220 (55.6)C	30,875 (62.7)D	<0.001*
High risk	780 (1.0)	1314 (2.8)	2588 (4.5)	4323 (7.3)	<0.001*	2314 (1.2)a	4449 (2.9)	6496 (5.5)C	4432 (9.0)D	<0.001*
Both sarcopenia and frailty	2081 (2.8)	2597 (5.5)	4237 (7.4)	5377 (9.1)	<0.001*	7800 (3.9)A	12,312 (7.9)B	13,625 (11.4)C	6707 (13.6)D	<0.001*

*Note*: “A” comparing <50 GMP versus <50 AUD *p* < 0.001 either for *z*‐test of proportions or *t*‐test for normally distributed data, Mann–Whitney test for non‐parametric data. “B” comparing 50–60 GMP versus 50–60 AUD *p* < 0.001. “C” comparing 61–70 GMP versus 61–70 AUD *p* < 0.001. “D” comparing 71–80 GMP versus 71–80 AUD *p* < 0.001. “a” comparing <50 GMP versus <50 AUD *p* < 0.05. “b” comparing 50–60 GMP versus 50–60 AUD *p* < 0.05. “c” comparing 61–70 GMP versus 61–70 AUD *p* < 0.05. “d” comparing 71–80 GMP versus 71–80 AUD *p* < 0.05.

* Post hoc analysis of ANOVA with Tukey HSD reveals statistically significant differences among all groups within either GMP or AUD groups.

Patients were then stratified into cohorts of those with sarcopenia, those with frailty, and those with both sarcopenia and frailty across the lifespan with and without AUD. Characteristics of patients with sarcopenia with and without AUD across the lifespan were examined in Table 2. Across all age ranges in patients with sarcopenia, there was a higher percentage of male patients with AUD as compared to those without AUD. For those with sarcopenia, frailty was also higher across all age groups in patients with AUD versus those without which increased across age groups. Patients with sarcopenia with and without AUD had similar numbers of comorbidities as examined by the Elixhauser comorbidity score. Total hospital charges and length of stay were significantly greater across all age groups for those with sarcopenia and AUD compared to those without AUD.

**TABLE 2:** Demographic and clinical characteristics of hospitalized patients with sarcopenia without/with alcohol use disorder.

Age categories in years	Patients without AUD (*n* = 19,664)					Patients with AUD (*n* = 56,050)				
	<50 (*n* = 3464)	50–60 (*n* = 3814)	61–70 (*n* = 5676)	71–80 (*n* = 6710)	*p*‐Value	<50 (*n* = 12,581)	50–60 (*n* = 17,491)	61–70 (*n* = 17,895)	71–80 (*n* = 8083)	*p*‐Value
Age (mean (SD))	38.31 (8.73)	56.09 (2.76)	65.63 (2.82)	75.88 (3.11)	<0.001*	40.92 (7.25)A	56.02 (2.75)	65.03 (2.78)C	74.82 (2.98)D	<0.001*
Female (%)	1723 (49.7)	1784 (46.8)	2604 (45.9)	3510 (52.3)	<0.001	5041 (40.1)A	5906 (33.8)B	5053 (28.2)C	2419 (29.9)D	<0.001*
Race (%)										
White	1856 (54.9)	2362 (63.1)	3627 (65.1)	4608 (69.9)	<0.001*	7398 (60.4)A	11,005 (64.3)	11,732 (66.9)c	5737 (72.4)d	<0.001*
Black	780 (23.1)	840 (22.4)	1130 (20.3)	1030 (15.6)	<0.001	2432 (19.9)A	4032 (23.5)	4091 (23.3)C	1369 (17.3)d	<0.001
Other	745 (22.0)	541 (14.5)	817 (14.7)	950 (14.4)	<0.001	2413 (19.7)a	2089 (12.2)B	1719 (9.8)C	813 (10.3)D	<0.001
Mean household income by zip code (%)										
0–25th percentile	1250 (37.1)	1331 (35.8)	1873 (33.7)	1928 (29.2)	<0.001	4415 (36.5)	6800 (40.3)B	6604 (38.2)C	2477 (31.3)d	<0.001*
26–50th percentile	900 (26.7)	1002 (26.9)	1415 (25.5)	1741 (26.4)	<0.001	3186 (26.4)	4448 (26.4)	4433 (25.6)	2044 (25.8)	<0.001
51st to 75th percentile	697 (20.7)	796 (21.4)	1342 (24.1)	1584 (24.0)	<0.001	2674 (22.1)	3539 (21.0)	3738 (21.6)C	1827 (23.1)	<0.001
76–100th percentile	518 (15.4)	589 (15.8)	928 (16.7)	1352 (20.5)	<0.001	1811 (15.0)	2077 (12.3)B	2526 (14.6)C	1568 (19.8)	<0.001
Urban–rural classification for US county (%)										
Central counties of metro areas of ≥1 million	1234 (36.3)	1283 (34.0)	1919 (34.1)	2197 (32.8)	<0.001	4499 (36.7)	5868 (34.3)	6034 (34.3)	2617 (32.6)	<0.001
Fringe counties of metro areas of ≥1 million	741 (21.8)	854 (22.7)	1278 (22.7)	1626 (24.3)	<0.001	2561 (20.9)	3518 (20.6)b	3637 (20.7)C	1911 (23.8)	<0.001
Counties in metro areas of 250,000–999,999	701 (20.6)	808 (21.4)	1197 (21.3)	1358 (20.3)	<0.001	2684 (21.9)	3859 (22.6)	3908 (22.2)	1741 (21.7)	<0.001
Counties in metro areas of 50,000–249,999	320 (9.4)	364 (9.7)	526 (9.3)	644 (9.6)	<0.001	1169 (9.5)	1757 (10.3)	1807 (10.3)	848 (10.6)	<0.001
Micropolitan counties	250 (7.3)	282 (7.5)	451 (8.0)	535 (8.0)	<0.001	840 (6.9)	1306 (7.6)	1370 (7.8)	574 (7.1)d	<0.001
Not metropolitan or micropolitan counties	157 (4.6)	177 (4.7)	259 (4.6)	333 (5.0)	<0.001	490 (4.0)	782 (4.6)	812 (4.6)	338 (4.2)d	<0.001
Primary payer for insurance (%)										
Medicare	612 (17.7)	1151 (30.2)	3769 (66.4)	6046 (90.2)	<0.001*	1334 (10.6)A	3760 (21.5)B	10,548 (59.0)C	7268 (90.0)	<0.001*
Medicaid	1458 (42.1)	1213 (31.9)	646 (11.4)	118 (1.8)	<0.001*	6102 (48.6)A	7578 (43.4)B	3230 (18.1)C	94 (1.2)d	<0.001*
Private insurance	946 (27.3)	1096 (28.8)	1006 (17.7)	398 (5.9)	<0.001	2709 (21.6)A	3698 (21.2)B	2639 (14.8)C	453 (5.6)	<0.001
Other	444 (12.8)	347 (9.1)	251 (4.4)	141 (2.1)	<0.001*	2420 (19.3)A	2429 (13.9)B	1459 (8.2)C	263 (3.3)D	<0.001*
Comorbidities (%)										
CHF	296 (8.5)	586 (15.4)	1249 (22.0)	1965 (29.3)	<0.001*	799 (6.4)A	2050 (11.7)B	3165 (17.7)C	1938 (24.0)D	<0.001*
COPD	215 (6.2)	959 (25.1)	1799 (31.7)	2058 (30.7)	<0.001	1049 (8.3)A	5151 (29.4)B	6970 (38.9)C	3351 (41.5)D	<0.001*
Alpha 1 antitrypsin deficiency	150 (4.3)	162 (4.2)	200 (3.5)	214 (3.2)	0.006	683 (5.4)	953 (5.4)	789 (4.4)	259 (3.2)	<0.001
CKD	427 (12.3)	712 (18.7)	1396 (24.6)	2003 (29.9)	<0.001*	944 (7.5)A	2025 (11.6)B	2521 (14.1)C	1558 (19.3)D	<0.001*
Type II DM	503 (14.5)	1013 (26.6)	1793 (31.6)	2234 (33.3)	<0.001*	1662 (13.2)a	2990 (17.1)B	3125 (17.5)C	1459 (18.1)D	<0.001
Anemia	484 (14.0)	609 (16.0)	1003 (17.7)	1107 (16.5)	<0.001	1291 (10.3)A	2097 (12.0)B	2081 (11.6)C	931 (11.5)D	<0.001
Elixhauser comorbidity score	27.80 (12.16)	29.93 (12.34)	30.82 (12.30)	31.38 (12.53)	<0.001*	24.60 (12.19)A	26.26 (12.35)B	26.78 (12.28)C	27.41 (12.42)D	<0.001*
Clinical outcomes										
Disposition of patient upon discharge (%)										
Routine discharge	2018 (60.7)	1743 (48.9)	1896 (36.0)	1496 (24.4)	<0.001*	8213 (68.0)A	9232 (55.8)B	6996 (41.5)C	1944 (25.6)d	<0.001*
Transfer to short‐term hospital	113 (3.4)	119 (3.3)	164 (3.1)	150 (2.4)	<0.001	395 (3.3)	469 (2.8)	462 (2.7)	179 (2.4)	<0.001
Transfer to other: SNF, ICF	496 (14.9)	842 (23.6)	1803 (34.2)	2765 (45.0)	<0.001*	1496 (12.4)A	3559 (21.5)b	5794 (34.4)	3603 (47.5)D	<0.001*
Transfer to Home Health Care (HHC)	531 (16.0)	778 (21.8)	1345 (25.5)	1703 (27.7)	<0.001*	1212 (10.0)A	2598 (15.7)B	3184 (18.9)C	1750 (23.1)D	<0.001*
Against medical advice (AMA)	169 (5.1)	86 (2.4)	65 (1.2)	26 (0.4)	759 (6.3)	679 (4.1)	408 (2.4)	110 (1.5)	169 (5.1)	86 (2.4)
Total charges in $ (median (IQR))	28,355 [16,195, 52,745]	35,571 [20,015, 67,508]	38,233 [21,409, 71,785]	38,146 [21,570, 70,930]	<0.001	44,195 [24,097, 91,338]A	47,113 [26,052, 91,572]B	49,628 [27,589, 95,515]C	51,365 [28,570, 98,137]D	<0.001
Length of Stay in days (median (IQR))	3.0 [2.0, 5.0]	3.0 [2.0, 6.0]	4.0 [2.0, 6.0]	4.0 [2.0, 6.0]	<0.001	5.0 [3.0, 9.0]	5.0 [3.0, 9.0]	5.0 [3.0, 10.0]	6.0 [3.0, 9.0]	<0.001
In‐hospital mortality (%)	136 (3.9)	242 (6.4)	402 (7.1)	564 (8.4)	<0.001	498 (4.0)	947 (5.4)b	1036 (5.8)C	493 (6.1)D	<0.001
Number of ICD‐10‐CM coded on discharge record (mean (SD))	17.43 (6.69)	19.06 (6.32)	20.27 (6.34)	20.61 (6.22)	<0.001*	18.24 (6.23)A	19.54 (6.22)B	20.20 (6.13)	21.09 (6.16)D	<0.001*
SDOHr (%)	24,662 (13.2)	14,306 (10.4)	6505 (6.4)	1130 (2.7)	<0.001*	1164 (9.3)A	1515 (8.7)B	1189 (6.6)	293 (3.6)D	<0.001*
Frailty (%)										
Low risk	1383 (39.9)	1217 (31.9)	1439 (25.4)	1333 (19.9)	<0.001*	4781 (38.0)a	5179 (29.6)b	4270 (23.9)c	1376 (17.0)D	<0.001*
Intermediate risk	1938 (55.9)	2334 (61.2)	3646 (64.2)	4419 (65.9)	<0.001*	7322 (58.2)a	11,196 (64.0)b	11,977 (66.9)C	5563 (68.8)D	<0.001*
High risk	143 (4.1)	263 (6.9)	591 (10.4)	958 (14.3)	<0.001*	478 (3.8)	1116 (6.4)	1648 (9.2)c	1144 (14.2)	<0.001*
Both sarcopenia and frailty	2081 (60.1)	2597 (68.1)	4237 (74.6)	5377 (80.1)	<0.001*	7800 (62.0)a	12,312 (70.4)b	13,625 (76.1)c	6707 (83.0)D	<0.001*

*Note*: “A” comparing <50 GMP versus <50 AUD *p* < 0.001. “B” comparing 50–60 GMP versus 50–60 AUD *p* < 0.001. “C” comparing 61–70 GMP versus 61–70 AUD *p* < 0.001. “D” comparing 71–80 GMP versus 71–80 AUD *p* < 0.001. “a” comparing <50 GMP versus <50 AUD *p* < 0.05. “b” comparing 50–60 GMP versus 50–60 AUD *p* < 0.05. “c” comparing 61–70 GMP versus 61–70 AUD *p* < 0.05. “d” comparing 71–80 GMP versus 71–80 AUD *p* < 0.05.

Abbreviations: CHF, congestive heart failure; CKD, chronic kidney disease; COPD, chronic obstructive pulmonary disease; ICF, intermediate care facility; SDOH, social determinants of health; SDOHr, social determinants of health risk factor; SNF, skilled nursing facility; Type II DM, type II diabetes mellitus.

* Post hoc analysis of ANOVA with Tukey HSD reveals statistically significant differences among all groups within either GMP or AUD groups.

Characteristics of patients with frailty with and without AUD across age groups were examined in Table 3. There was a higher number of female patients with frailty across all age ranges in patients without AUD as compared to those with AUD. Sarcopenia (muscle loss phenotype) was higher across all age groups in the AUD patients versus patients without AUD. Mortality was higher in patients with AUD and frailty as compared to those without AUD except for patients aged 71–80. Patients with AUD and frailty also had greater numbers of SDOHr, but had similar Elixhauser comorbidity scores as compared to patients without AUD.

**TABLE 3:** Demographic and clinical characteristics of hospitalized patients with frailty without/with alcohol use disorder.

Age categories in years	Patients without AUD (*n* = 111,551)					Patients with AUD (*n* = 254,861)				
	<50 (*n* = 21,516)	50–60 (*n* = 21,186)	61–70 (*n* = 31,081)	71–80 (*n* = 37,768)	*p* Value	<50 (*n* = 69,584)	50–60 (*n* = 77,254)	61–70 (*n* = 72,716)	71–80 (*n* = 35,307)	*p* Value
Age (mean (SD))	38.51 (8.60)	55.97 (2.79)	65.63 (2.86)	75.83 (3.14)	<0.001*	39.87 (7.82)A	55.87 (2.77)B	64.99 (2.81)C	74.90 (3.01)D	<0.001*
Female (%)	10,306 (47.9)	9677 (45.7)	14,917 (48.0)	19,906 (52.7)	<0.001	24,711 (35.5)A	23,051 (29.8)B	19,093 (26.3)C	9647 (27.3)D	<0.001
Race (%)										
White	11,108 (52.7)	12,577 (60.6)	20,344 (66.8)	27,317 (73.8)	<0.001*	39,261 (57.8)A	48,152 (63.5)B	48,967 (68.6)C	26,673 (76.9)D	<0.001*
Black	5086 (24.1)	4663 (22.5)	5691 (18.7)	4633 (12.5)	<0.001*	13,744 (20.2)A	17,029 (22.5)	14,560 (20.4)C	4580 (13.2)d	<0.001*
Other	4870 (23.1)	3502 (16.9)	4438 (14.6)	5062 (13.7)	<0.001*	14,943 (22.0)A	10,625 (14.0)B	7862 (11.0)C	3439 (9.9)D	<0.001*
Mean household income by zip code (%)										
0–25th percentile	7835 (37.4)	7531 (36.5)	10,053 (32.9)	10,457 (28.1)	<0.001	24,452 (36.5)A	29,056 (39.1)B	25,459 (36.1)C	9871 (28.5)	<0.001*
26–50th percentile	5569 (26.6)	5485 (26.6)	8196 (26.8)	9918 (26.6)	0.857	17,527 (26.2)a	19,290 (25.9)B	18,454 (26.2)C	8944 (25.8)d	0.292
51st to 75th percentile	4514 (21.5)	4503 (21.8)	7129 (23.4)	9114 (24.5)	<0.001	14,923 (22.3)	15,634 (21.0)b	15,503 (22.0)C	8397 (24.3)	<0.001*
76–100th percentile	3054 (14.6)	3137 (15.2)	5152 (16.9)	7730 (20.8)	<0.001	10,036 (15.0)	10,356 (13.9)B	11,047 (15.7)C	7390 (21.4)	<0.001*
Urban–rural classification for US county (%)										
Central counties of metro areas of ≥1 million	7319 (34.5)	6774 (32.3)	9464 (30.6)	10,518 (27.9)	<0.001*	24,080 (35.5)	25,763 (34.2)B	23,400 (32.7)C	10,766 (30.7)D	<0.001*
Fringe counties of metro areas of ≥1 million	4719 (22.3)	4893 (23.3)	7273 (23.5)	9583 (25.4)	<0.001	15,086 (22.2)	16,433 (21.8)B	15,909 (22.3)C	8765 (25.0)	<0.001
Counties in metro areas of 250,000–999,999	4735 (22.3)	4639 (22.1)	6769 (21.9)	8330 (22.1)	0.7110	15,197 (22.4)	17,059 (22.6)	16,106 (22.5)	7884 (22.5)	0.7510
Counties in metro areas of 50,000–249,999	1928 (9.1)	1932 (9.2)	3036 (9.8)	3823 (10.1)	<0.001	6279 (9.3)	7189 (9.5)	7058 (9.9)	3511 (10.0)	<0.001
Micropolitan counties	1563 (7.4)	1635 (7.8)	2606 (8.4)	3267 (8.7)	<0.001	4502 (6.6)A	5552 (7.4)b	5636 (7.9)C	2579 (7.4)D	<0.001
Not metropolitan or micropolitan counties	938 (4.4)	1085 (5.2)	1756 (5.7)	2164 (5.7)	<0.001	2724 (4.0)a	3386 (4.5)B	3388 (4.7)C	1583 (4.5)D	<0.001
Primary payer for insurance (%)										
Medicare	4087 (19.0)	6959 (32.9)	20,692 (66.7)	33,995 (90.1)	<0.001*	6828 (9.8)A	17,084 (22.2)B	42,296 (58.2)C	31,634 (89.7)	<0.001*
Medicaid	8185 (38.1)	6055 (28.6)	3116 (10.0)	607 (1.6)	<0.001*	31,227 (45.0)A	30,331 (39.3)B	11,758 (16.2)C	382 (1.1)D	<0.001*
Private insurance	5871 (27.3)	6010 (28.4)	5667 (18.3)	2287 (6.1)	<0.001*	16,018 (23.1)A	18,301 (23.7)B	12,501 (17.2)C	2151 (6.1)	<0.001
Other	3332 (15.5)	2134 (10.1)	1568 (5.1)	847 (2.2)	<0.001*	15,367 (22.1)A	11,410 (14.8)B	6063 (8.3)C	1114 (3.2)D	<0.001*
Comorbidities (%)										
CHF	2538 (11.8)	4692 (22.1)	8798 (28.3)	12,674 (33.6)	<0.001*	5723 (8.2)A	12,524 (16.2)B	16,277 (22.4)C	9660 (27.4)D	<0.001*
COPD	1430 (6.6)	4852 (22.9)	9107 (29.3)	11,139 (29.5)	<0.001	4775 (6.9)	19,755 (25.6)b	25,146 (34.6)C	12,693 (36.0)D	<0.001*
Alpha 1 antitrypsin deficiency	527 (2.4)	553 (2.6)	684 (2.2)	648 (1.7)	<0.001	2505 (3.6)A	2690 (3.5)B	2117 (2.9)C	753 (2.1)d	<0.001*
CKD	4194 (19.5)	6025 (28.4)	10,495 (33.8)	14,341 (38.0)	<0.001*	6709 (9.6)A	12,158 (15.7)B	13,947 (19.2)C	8794 (24.9)D	<0.001*
Type II DM	4754 (22.1)	8562 (40.4)	13,833 (44.5)	16,513 (43.7)	<0.001	10,177 (14.6)	18,076 (23.4)B	17,595 (24.2)C	8685 (24.6)	<0.001
Anemia	2487 (11.6)	2878 (13.6)	4350 (14.0)	4837 (12.8)	<0.001	4256 (6.1)A	6325 (8.2)B	6068 (8.3)	2994 (8.5)	<0.001
Elixhauser comorbidity score	25.52 (12.04)	28.01 (12.31)	29.27 (12.44)	29.64 (12.48)	<0.001	21.91 (12.04)A	24.27 (12.35)B	24.96 (12.39)C	25.28 (12.47)D	<0.001
Clinical outcomes										
Disposition of patient upon discharge (%)										
Routine discharge	14,277 (68.5)	11,708 (57.7)	13,662 (46.3)	12,722 (35.8)	<0.001*	48,350 (72.4)A	45,403 (62.0)B	34,068 (49.4)C	11,899 (35.5)	<0.001*
Transfer to short‐term hospital	642 (3.1)	623 (3.1)	947 (3.2)	945 (2.7)	<0.001	2072 (3.1)	2194 (3.0)	2078 (3.0)	870 (2.6)	<0.001
Transfer to other: SNF, ICF	2481 (11.9)	3908 (19.3)	8292 (28.1)	13,111 (36.9)	<0.001*	7242 (10.8)A	12,861 (17.5)B	19,263 (27.9)	12,991 (38.7)D	<0.001*
Transfer to Home Health Care (HHC)	2240 (10.8)	3500 (17.2)	6219 (21.1)	8563 (24.1)	<0.001*	4330 (6.5)A	9370 (12.8)B	11,555 (16.8)C	7305 (21.8)D	<0.001*
Against medical advice (AMA)	1192 (5.7)	562 (2.8)	398 (1.3)	213 (0.6)	<0.001*	4790 (7.2)A	3458 (4.7)B	1973 (2.9)C	468 (1.4)D	<0.001*
Total charges in $ (median (IQR))	44,195 [24,097, 91,338]	47,113 [26,052, 91,572]	49,628 [27,589, 95,515]	51,365 [28,570, 98,137]	<0.001	38,755 [21,686, 76,623]A	42,815 [23,899, 82,298]B	45,692 [25,439, 86,378]C	45,754 [25,867, 85,411]D	<0.001
Length of stay in days (median (IQR))	5.0 [3.0, 9.0]	5.0 [3.0, 9.0]	5.00 [3.0, 10.0]	6.0 [3.0, 9.0]	<0.001	4.0 [2.0, 7.0]	4.0 [2.0, 7.0]	4.0 [3.0, 8.0]	4.0 [3.0, 8.0]	<0.001
In‐hospital mortality (%)	671 (3.1)	870 (4.1)	1551 (5.0)	2194 (5.8)	<0.001*	2739 (3.9)	3935 (5.1)	3729 (5.1)	1752 (5.0)	<0.001
Number of ICD‐10‐CM on discharge (mean (SD))	16.36 (6.22)	18.36 (6.06)	19.08 (6.06)	19.33 (6.02)	<0.001	16.85 (5.86)A	18.52 (5.95)B	19.28 (5.98)C	19.80 (5.95)D	<0.001
SDOHr (%)	1200 (5.6)	922 (4.4)	657 (2.1)	430 (1.1)	<0.001*	7112 (10.2)A	6989 (9.0)	4413 (6.1)	1043 (3.0)	<0.001*
Sarcopenia (%)	2081 (9.7)	2597 (12.3)	4237 (13.6)	5377 (14.2)	<0.001*	7800 (11.2)A	12,312 (15.9)	13,625 (18.7)C	6707 (19.0)	<0.001*
Frailty (%)										
Intermediate risk	20,736 (96.4)	19,872 (93.8)	28,493 (91.7)	33,445 (88.6)		67,270 (96.7)	72,805 (94.2)	66,220 (91.1)	30,875 (87.4)	
High risk	780 (3.6)	1314 (6.2)	2588 (8.3)	4323 (11.4)		2314 (3.3)	4449 (5.8)	6496 (8.9)	4432 (12.6)	
Both sarcopenia and frailty	2081 (9.7)	2597 (12.3)	4237 (13.6)	5377 (14.2)	<0.001*	7800 (11.2)A	12,312 (15.9)	13,625 (18.7)C	6707 (19.0)D	<0.001*

*Note*: “A” comparing <50 GMP versus <50 AUD *p* < 0.001. “B” comparing 50–60 GMP versus 50–60 AUD *p* < 0.001. “C” comparing 61–70 GMP versus 61–70 AUD *p* < 0.001. “D” comparing 71–80 GMP versus 71–80 AUD *p* < 0.001. “a” comparing <50 GMP versus <50 AUD *p* < 0.05. “b” comparing 50–60 GMP versus 50–60 AUD *p* < 0.05. “c” comparing 61–70 GMP versus 61–70 AUD *p* < 0.05. “d” comparing 71–80 GMP versus 71–80 AUD *p* < 0.05.

Abbreviations: CHF, congestive heart failure; CKD, chronic kidney disease; COPD, chronic obstructive pulmonary disease; ICF, intermediate care facility; SDOHr, social determinants of health risk factor; SNF, skilled nursing facility; Type II DM, type II diabetes mellitus.

* Post hoc analysis of ANOVA with Tukey HSD reveals statistically significant differences among all groups within either GMP or AUD groups.

When examining patients with both sarcopenia and frailty with and without AUD across age groups (Table 4), there was a higher percentage of female patients without AUD as compared to patients with AUD. In‐hospital mortality percentages were higher in patients <50 with AUD but were lower across all other age groups in patients with AUD as compared to those without AUD. SDOHr were again higher among the AUD group versus the non‐AUD group, and comorbidity scores were similar across both groups and age ranges.

**TABLE 4:** Demographic and clinical characteristics of hospitalized patients with sarcopenia+frailty without/with alcohol use disorder.

Age categories in years	Patients without AUD (*n* = 14,292)					Patients with AUD (*n* = 40,444)				
	<50 (*n* = 2081)	50–60 (*n* = 2597)	61–70 (*n* = 4237)	71–80 (*n* = 5377)	*p* Value	<50 (*n* = 7800)	50–60 (*n* = 12,312)	61–70 (*n* = 13,625)	71–80 (*n* = 6707)	*p* Value
Age (mean (SD))	38.95 (8.52)	56.15 (2.76)	65.69 (2.81)	75.93 (3.10)	<0.001*	41.35 (7.06)A	56.12 (2.73)	65.10 (2.78)C	74.90 (2.99)D	<0.001*
Female (%)	1037 (49.8)	1222 (47.1)	1964 (46.4)	2797 (52.0)	<0.001	3347 (42.9)A	4353 (35.4)B	4004 (29.4)C	2041 (30.4)D	<0.001
Race (%)										
White	1080 (53.2)	1595 (62.5)	2664 (64.0)	3625 (68.7)	<0.001*	4566 (60.2)A	7867 (65.3)b	9074 (67.9)C	4763 (72.5)D	<0.001*
Black	497 (24.5)	603 (23.6)	892 (21.4)	859 (16.3)	<0.001*	1492 (19.7)A	2773 (23.0)	3020 (22.6)	1143 (17.4)	<0.001
Other	453 (22.3)	352 (13.8)	605 (14.5)	789 (15.0)	<0.001	1531 (20.2)a	1410 (11.7)b	1271 (9.5)C	665 (10.1)D	<0.001*
Mean household income by zip code (%)										
0–25th percentile	785 (38.8)	886 (35.1)	1399 (33.7)	1548 (29.3)	<0.001*	2734 (36.5)a	4756 (40.0)B	4934 (37.4)C	2042 (31.1)d	<0.001
26–50th percentile	531 (26.3)	692 (27.4)	1057 (25.4)	1397 (26.4)	<0.001	1963 (26.2)	3093 (26.0)	3359 (25.5)	1694 (25.8)	<0.001
51st to 75th percentile	413 (20.4)	553 (21.9)	1009 (24.3)	1270 (24.0)	<0.001	1674 (22.4)	2529 (21.3)	2906 (22.0)C	1520 (23.1)	<0.001
76–100th percentile	293 (14.5)	396 (15.7)	690 (16.6)	1077 (20.4)	<0.001	1118 (14.9)	1519 (12.8)B	1999 (15.1)c	1320 (20.1)	<0.001
Urban–rural classification for US county (%)										
Central counties of metro areas of ≥1 million	732 (35.8)	874 (34.1)	1435 (34.1)	1760 (32.8)	0.234	2829 (37.3)	4099 (34.0)	4533 (33.9)	2172 (32.6)	<0.001
Fringe counties of metro areas of ≥1 million	440 (21.5)	577 (22.5)	973 (23.1)	1322 (24.7)	0.006	1589 (20.9)	2483 (20.6)b	2840 (21.2)C	1614 (24.2)	<0.001
Counties in metro areas of 250,000–999,999	440 (21.5)	565 (22.0)	889 (21.1)	1093 (20.4)	0.510	1674 (22.1)	2699 (22.4)	2969 (22.2)	1442 (21.6)	0.842
Counties in metro areas of 50,000–249,999	179 (8.8)	250 (9.7)	382 (9.1)	496 (9.3)	0.662	706 (9.3)	1267 (10.5)	1388 (10.4)	706 (10.6)d	0.010
Micropolitan counties	154 (7.5)	186 (7.3)	326 (7.7)	429 (8.0)	0.596	498 (6.6)	943 (7.8)	1042 (7.8)	460 (6.9)d	<0.001
Not metropolitan or micropolitan counties	100 (4.9)	113 (4.4)	202 (4.8)	261 (4.9)	0.789	295 (3.9)	556 (4.6)	618 (4.6)	272 (4.1)d	0.0258
Primary payer for insurance (%)										
Medicare	432 (20.8)	848 (32.7)	2870 (67.8)	4840 (90.1)	<0.001*	863 (11.1)A	2781 (22.6)B	8203 (60.3)C	6062 (90.4)	<0.001*
Medicaid	886 (42.7)	808 (31.2)	479 (11.3)	96 (1.8)	<0.001*	3844 (49.3)A	5299 (43.1)B	2366 (17.4)C	70 (1.0)D	<0.001*
Private insurance	517 (24.9)	710 (27.4)	721 (17.0)	321 (6.0)	<0.001	1685 (21.6)a	2650 (21.6)B	1974 (14.5)C	365 (5.4)	<0.001
Other	242 (11.7)	226 (8.7)	164 (3.9)	115 (2.1)	<0.001*	1400 (18.0)A	1562 (12.7)B	1070 (7.9)C	206 (3.1)d	<0.001*
Comorbidities (%)										
CHF	237 (11.4)	450 (17.3)	1013 (23.9)	1650 (30.7)	<0.001*	577 (7.4)A	1537 (12.5)B	2525 (18.5)C	1633 (24.3)D	<0.001*
COPD	133 (6.4)	647 (24.9)	1283 (30.3)	1617 (30.1)	<0.001	684 (8.8)A	3638 (29.5)B	5239 (38.5)C	2769 (41.3)D	<0.001*
Alpha 1 antitrypsin deficiency	117 (5.6)	132 (5.1)	161 (3.8)	188 (3.5)	<0.001	477 (6.1)	708 (5.8)	649 (4.8)	222 (3.3)	<0.001*
CKD	366 (17.6)	621 (23.9)	1263 (29.8)	1829 (34.0)	<0.001*	853 (10.9)A	1859 (15.1)B	2326 (17.1)C	1462 (21.8)D	<0.001*
Type II DM	362 (17.4)	720 (27.7)	1415 (33.4)	1845 (34.3)	0.06	1087 (13.9)A	2206 (17.9)B	2473 (18.2)C	1237 (18.4)D	0.018
Anemia	371 (17.8)	476 (18.3)	844 (19.9)	964 (17.9)	<0.001	956 (12.3)A	1680 (13.6)B	1773 (13.0)C	836 (12.5)D	<0.001
Elixhauser comorbidity score	29.47 (12.30)	30.98 (12.17)	31.66 (12.30)	31.94 (12.54)	<0.001*	25.94 (12.17)A	27.04 (12.39)B	27.47 (12.27)C	27.85 (12.40)D	<0.001*
Clinical outcomes										
Disposition of patient upon discharge (%)										
Routine discharge	1014 (51.9)	986 (41.3)	1135 (29.4)	963 (19.9)	<0.001*	4451 (60.9)A	5582 (48.9)B	4492 (35.5)C	1373 (22.0)D	<0.001*
Transfer to short‐term hospital	83 (4.2)	82 (3.4)	125 (3.2)	117 (2.4)	<0.001	289 (4.0)	357 (3.1)	359 (2.8)	161 (2.6)	<0.001
Transfer other: SNF, ICF	404 (20.7)	690 (28.9)	1547 (40.0)	2415 (49.9)	<0.001*	1228 (16.8)A	3024 (26.5)b	5021 (39.7)	3208 (51.4)d	<0.001*
Transfer to Home Health Care (HHC)	364 (18.6)	570 (23.9)	1019 (26.4)	1327 (27.4)	<0.001	909 (12.4)A	1994 (17.5)B	2478 (19.6)C	1424 (22.8)D	<0.001*
Against medical advice (AMA)	88 (4.5)	61 (2.6)	41 (1.1)	17 (0.4)	<0.001*	431 (5.9)a	452 (4.0)B	291 (2.3)C	73 (1.2)D	<0.001*
Total charges in $ (median (IQR))	38,755 [21,686, 76,623]	42,815 [23,899, 82,298]	45,692 [25,439, 86,378]	45,754 [25,867, 85,411]	<0.001	38,755 [21,686, 76,623]	42,815 [23,899, 82,298]	45,692 [25,439, 86,378]	45,754 [25,867, 85411.00]	<0.001*
Length of stay in days (median (IQR))	4.0 [2.0, 7.0]	4.0 [2.0, 7.0]	4.0 [3.0, 8.0]	4.0 [3.0, 8.0]	<0.001	4.00 [2.0, 7.0]	4.0 [2.0, 7.0]	4.0 [3.0, 8.0]	4.0 [3.0, 8.0]	<0.001
In‐hospital mortality (%)	127 (6.1)	204 (7.9)	369 (8.7)	532 (9.9)	<0.001	487 (6.2)	897 (7.3)	971 (7.1)C	464 (6.9)D	0.033
Number of ICD‐10‐CM on discharge (mean (SD))	20.23 (6.01)	21.02 (5.82)	21.79 (5.94)	21.82 (5.89)	<0.001*	20.65 (5.85)a	21.33 (5.89)b	21.59 (5.84)c	22.09 (5.89)d	<0.001*
SDOHr (%)	125 (6.0)	112 (4.3)	97 (2.3)	84 (1.6)	<0.001*	678 (8.7)A	1035 (8.4)B	894 (6.6)C	246 (3.7)D	<0.001*

*Note*: “A” comparing <50 GMP versus <50 AUD *p* < 0.001. “B” comparing 50–60 GMP versus 50–60 AUD *p* < 0.001. “C” comparing 61–70 GMP versus 61–70 AUD *p* < 0.001. “D” comparing 71–80 GMP versus 71–80 AUD *p* < 0.001. “a” comparing <50 GMP versus <50 AUD *p* < 0.05. “b” comparing 50–60 GMP versus 50–60 AUD *p* < 0.05. “c” comparing 61–70 GMP versus 61–70 AUD *p* < 0.05. “d” comparing 71–80 GMP versus 71–80 AUD *p* < 0.05.

Abbreviations: CHF, congestive heart failure; CKD, chronic kidney disease; COPD, chronic obstructive pulmonary disease; ICF, intermediate care facility; SDOHr, social determinants of health risk factor; SNF, skilled nursing facility; Type II DM, type II diabetes mellitus.

* Post hoc analysis of ANOVA with Tukey HSD reveals statistically significant differences among all groups within either GMP or AUD groups.

Regression models were generated across different age groups comparing outcomes in AUD versus GMP as shown in Table 5. For almost all age categories, there was an independent effect of AUD associated with adverse clinical outcomes, including sarcopenia, frailty, sarcopenia+frailty, and length of stay. When comparing AUD to the GMP, there was a greater odds of diagnosis with sarcopenia+frailty when compared to either sarcopenia alone or frailty alone across all age categories (AUD vs. GMP adjusted odds ratio [<50; 50–60; 61–70; 71–80]: 1.68 (CI: 1.6–1.78), 1.71 (CI: 1.63–1.79), 1.86 (CI: 1.79–1.93), 1.81 (CI: 1.74–1.89)). There was also a greater odds for in‐hospital mortality when comparing AUD versus GMP across most age groups (AUD vs. GMP odds ratio [<50; 50–60; 61–70]: 1.55 (CI: 1.43–1.68); 1.41 (CI: 1.31–1.51); 1.21 (CI: 1.14–1.28)). However, in‐hospital mortality was not significantly different when comparing AUD versus GMP in those 71–80 years old (adj OR 0.95, CI: 0.89–1.02), which may represent a survivor bias. Routine discharge to home was less likely in those with AUD for age groups 61–70 years (adj OR 0.90, CI: 0.89–0.92) and 71–80 years (adj OR 0.79, CI: 0.78–0.81).

**TABLE 5:** Regression analysis comparing AUD versus GMP across age categories.

	AUD vs. GMP < 50 years		AUD vs. GMP 50–60 years		AUD vs. GMP 61–70 years		AUD vs. GMP 71–80 years	
	Unadj OR (95% CI)	Adj OR (95% CI)	Unadj OR (95% CI)	Adj OR (95% CI)	Unadj OR (95% CI)	Adj OR (95% CI)	Unadj OR (95% CI)	Adj OR (95% CI)
Sarcopenia	**1.40** **(1.34–1.45)**	**1.58** **(1.52–1.65)**	**1.43** **(1.38–1.48)**	**1.63** **(1.57–1.70)**	**1.60** **(1.55–1.66)**	**1.82** **(1.76–1.89)**	**1.54** **(1.49–1.60)**	**1.74** **(1.68–1.81)**
Frailty	**1.34** **(1.32–1.36)**	**1.47** **(1.44–1.50)**	**1.20** **(1.17–1.22)**	**1.35** **(1.32–1.38)**	**1.32** **(1.29–1.34)**	**1.51** **(1.48–1.54)**	**1.44** **(1.41–1.48)**	**1.63** **(1.59–1.68)**
Sarcopenia + frailty	**1.43** **(1.36–1.51)**	**1.68** **(1.60–1.77)**	**1.47** **(1.40–1.53)**	**1.71** **(1.63–1.79)**	**1.61** **(1.56–1.67)**	**1.86** **(1.79–1.93)**	**1.58** **(1.52–1.64)**	**1.81** **(1.74–1.89)**
CoH	**0.94** **(0.93–0.95)**	**0.96** **(0.95–0.96)**	**0.95** **(0.94–0.96)**	**0.97** **(0.96–0.98)**	**1.02** **(1.01–1.03)**	**1.05** **(1.04–1.06)**	**1.07** **(1.06–1.09)**	**1.10** **(1.09–1.12)**
LoS	**1.15** **(1.13–1.16)**	**1.18** **(1.16–1.19)**	**1.05** **(1.04–1.07)**	**1.09** **(1.08–1.11)**	**1.07** **(1.05–1.08)**	**1.11** **(1.09–1.13)**	**1.08** **(1.06–1.09)**	**1.12** **(1.11–1.14)**
In‐hospital mortality	**1.43** **(1.32–1.55)**	**1.55** **(1.43–1.68)**	**1.27** **(1.19–1.36)**	**1.41** **(1.31–1.51)**	**1.10** **(1.04–1.17)**	**1.21** **(1.14–1.28)**	**0.93** **(0.87–0.99)**	0.95 (0.89–1.02)
Routine discharge to home	**0.90** **(0.88–0.92)**	**0.89** **(0.87–0.91)**	**1.05** **(1.03–1.08)**	0.99 (0.97–1.01)	0.99 (0.97–1.01)	**0.90** **(0.89–0.92)**	**0.89** **(0.86–0.91)**	**0.79** **(0.78–0.81)**

*Note*: CoH and LoS represent log‐transformed linear regression models. Mortality and discharge to home represent logistic regression. Model adjusted for race, sex, and comorbidities as determined by Elixhauser comorbidity score. Bolded odds ratios are statistically significant.

**FIGURE 1:**
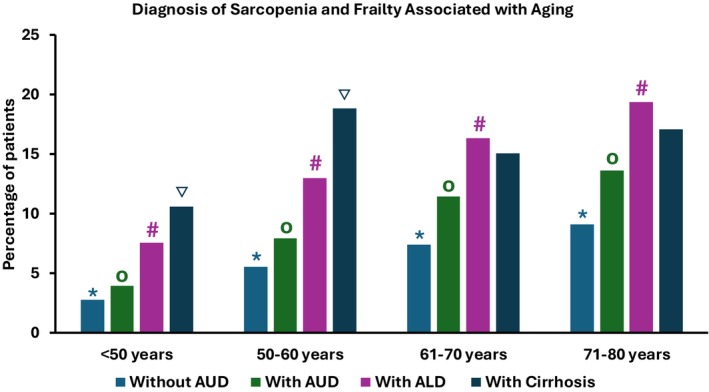
Sarcopenia and frailty with aging when comparing patients without alcohol use disorder (AUD), those with AUD, those with alcohol‐associated liver disease (ALD), and those with alcohol‐associated cirrhosis. A *z*‐test of proportions comparing each age group within each disease category was performed. * represents a statistically significant increase in *z*‐test of proportions with increasing age category for those without AUD. o represents a statistically significant increase in *z*‐test of proportions with increasing age category for those with AUD. # represents a statistically significant increase in *z*‐test of proportions with increasing age category for those with ALD. ▽ represents a statistically significant increase in *z*‐test of proportions with increasing age category for those with cirrhosis.

A sub‐analysis comparing patients with alcohol use disorder alone, AUD + alcohol‐associated liver disease (ALD), and AUD + alcohol‐associated cirrhosis was then performed (Table 6). Across all three groups, there was a higher percentage of younger patients (<50 age cohort) as well as male patients. SDOHr were significantly higher in those with AUD alone compared to those with AUD + ALD or AUD + alcohol‐associated cirrhosis. Interestingly, we found that those with AUD alone had the highest prevalence of COPD, but those with AUD + ALD or AUD + alcohol‐associated cirrhosis were more likely to have alpha 1 antitrypsin deficiency. We examined four main outcomes among patients with AUD alone, AUD + ALD, and AUD + alcohol‐associated cirrhosis; these outcomes included sarcopenia, frailty, sarcopenia + frailty, and mortality. When examining sarcopenia independently, patients with ALD or alcohol‐associated cirrhosis had a higher prevalence of sarcopenia as compared to patients with AUD across all age groups. Furthermore, sarcopenia prevalence increased in all age groups. When examining frailty independently, there were similar findings, with patients with ALD or cirrhosis having a higher rate than patients with AUD. When looking at sarcopenia and frailty, similar results were found, but there did not appear to be significant differences between patients with ALD and alcohol‐associated cirrhosis when examining sarcopenia and frailty. We then performed a *z*‐test of proportions comparing each age group within disease category and determined that those without AUD, those with AUD, and those with AUD + ALD showed a significant increase in sarcopenia+frailty prevalence based on increasing age group. For those with AUD + cirrhosis, there was an increase in prevalence of sarcopenia+frailty up until age 50–60 years, but the proportion was not significantly higher in those >60 which may represent a survival bias (Figure 1).

**TABLE 6:** Demographic and clinical characteristics of AUD alone versus alcohol‐associated liver disease.

Age categories in years	AUD alone (*n* = 434,959)					Alcohol‐associated liver disease (*n* = 15,411)					Alcohol‐associated cirrhosis (*n* = 15,411)				
	<50 (*n* = 168,535)	50–60 (*n* = 123,544)	61–70 (*n* = 98,936)	71–80 (*n* = 43,944)	*p*‐Value	<50 (*n* = 8426)	50–60 (*n* = 4234)	61–70 (*n* = 2220)	71–80 (*n* = 531)	*p*‐Value	<50 (*n* = 22,750)	50–60 (*n* = 27,610)	61–70 (*n* = 17,975)	71–80 (*n* = 4759)	*p*‐Value
Age (mean (SD))	37.92 (8.44)	55.71 (2.79)	64.90 (2.80)	74.86 (3.00)	<0.001*	38.36 (7.36)	55.43 (2.79)	64.47 (2.70)	74.20 (2.77)	<0.001*	42.23 (6.10)	55.61 (2.76)	64.59 (2.72)	74.32 (2.83)	<0.001*
Female (%)	51,777 (30.7)	31,765 (25.7)	23,489 (23.7)	11,598 (26.4)	<0.001	3459 (41.1)E	1548 (36.6)F	684 (30.8)G	168 (31.6)h	<0.001*	8382 (36.8)A	8542 (30.9)B	4756 (26.5)C	1143 (24.0)D	<0.001*
Race (%)															
White	93,728 (57.0)	74,822 (61.7)	65,925 (67.9)	33,369 (77.3)	<0.001	5109 (62.2)e	2820 (68.1)f	1482 (68.2)	415 (79.0)	<0.001*	13,312 (59.8)A	17,842 (65.7)B	11,976 (67.7)C	3402 (72.6)D	<0.001
Black	37,342 (22.7)	31,224 (25.7)	21,007 (21.6)	5707 (13.2)	<0.001*	1273 (15.5)E	811 (19.6)F	450 (20.7)	68 (13.0)	<0.001*	1853 (8.3)A	3145 (11.6)B	2430 (13.7)C	430 (9.2)D	<0.001
Other	33,472 (20.3)	15,232 (12.6)	10,198 (10.5)	4113 (9.5)	<0.001*	1830 (22.3)	512 (12.4)	240 (11.0)	42 (8.0)	<0.001*	7088 (31.9)A	6151 (22.7)B	3291 (18.6)C	854 (18.2)D	<0.001*
Mean household income by zip code (%)															
0–25th percentile	59,745 (36.9)	47,681 (40.2)	35,465 (37.1)	12,409 (28.9)	<0.001*	2331 (28.3)E	1319 (32.1)F	626 (29.0)G	124 (23.8)h	<0.001*	7255 (33.0)A	9529 (35.7)B	5881 (33.7)C	1303 (27.9)	<0.001
26–50th percentile	42,337 (26.2)	30,616 (25.8)	24,959 (26.1)	11,208 (26.1)	0.099	2177 (26.4)	1100 (26.8)F	592 (27.5)	132 (25.4)	0.806	5857 (26.6)a	7017 (26.3)b	4509 (25.8)	1185 (25.4)	0.391
51st to 75th percentile	35,257 (21.8)	24,061 (20.3)	20,733 (21.7)	10,399 (24.2)	<0.001	2118 (25.7)E	942 (23.0)F	532 (24.7)g	124 (23.8)	0.005*	5259 (23.9)A	5958 (22.3)B	4036 (23.1)C	1161 (24.9)	<0.001
76–100th percentile	24,528 (15.2)	16,165 (13.6)	14,533 (15.2)	8985 (20.9)	<0.001*	1610 (19.5)E	742 (18.1)F	405 (18.8)G	140 (26.9)h	<0.001*	3647 (16.6)A	4168 (15.6)B	3019 (17.3)C	1016 (21.8)	<0.001*
Urban–rural classification for US county (%)															
Central counties of metro areas of ≥1 million	57,955 (35.4)	41,752 (34.7)	31,571 (32.5)	13,279 (30.4)	<0.001*	2755 (33.1)e	1272 (30.6)F	712 (32.5)	151 (28.5)	0.007*	7840 (35.1)	9279 (34.3)	6118 (34.5)C	1511 (31.9)d	0.003
Fringe counties of metro areas of ≥1 million	37,051 (22.6)	26,563 (22.1)	21,653 (22.3)	10,767 (24.7)	<0.001	2043 (24.5)E	1040 (25.0)F	501 (22.9)	148 (28.0)	0.059	5017 (22.5)	6052 (22.4)	4047 (22.8)	1171 (24.7)	<0.001
Counties in metro areas of 250,000–999,999	35,703 (21.8)	26,620 (22.1)	21,796 (22.5)	9789 (22.4)	<0.001	1887 (22.7)e	952 (22.9)	473 (21.6)	107 (20.2)	0.440	5108 (22.9)A	6207 (22.9)B	3940 (22.2)	1058 (22.4)	0.507
Counties in metro areas of 50,000–249,999	15,095 (9.2)	11,114 (9.2)	9585 (9.9)	4338 (9.9)	<0.001	829 (10.0)e	387 (9.3)	232 (10.6)	58 (11.0)	0.271	1960 (8.8)	2529 (9.3)	1651 (9.3)c	482 (10.2)	0.005
Micropolitan counties	11,438 (7.0)	8780 (7.3)	7708 (7.9)	3353 (7.7)	<0.001	530 (6.4)	330 (7.9)	174 (8.0)	38 (7.2)	0.004*	1432 (6.4)a	1817 (6.7)b	1255 (7.1)C	313 (6.6)d	0.052
Not metropolitan or micropolitan counties	6657 (4.1)	5354 (4.5)	4771 (4.9)	2097 (4.8)	<0.001	282 (3.4)	182 (4.4)	96 (4.4)	27 (5.1)	0.009*	968 (4.3)a	1177 (4.3)	704 (4.0)C	198 (4.2)	0.273
Primary payer for insurance (%)															
Medicare	15,860 (9.4)	25,016 (20.3)	55,495 (56.2)	39,259 (89.4)	<0.001*	382 (4.5)e	645 (15.2)F	1105 (49.9)G	480 (90.4)H	<0.001*	1923 (8.5)A	5541 (20.1)B	9656 (53.8)C	4186 (88.0)D	<0.001*
Medicaid	71,662 (42.6)	47,068 (38.2)	16,301 (16.5)	503 (1.1)	<0.001*	3596 (42.8)E	1511 (35.7)F	391 (17.7)G	4 (0.8)H	<0.001*	11,455 (50.4)	11,632 (42.2)B	3403 (19.0)C	83 (1.7)d	<0.001*
Private insurance	40,940 (24.3)	30,628 (24.8)	18,015 (18.2)	2750 (6.3)	<0.001*	2403 (28.6)E	1329 (31.4)F	512 (23.1)G	35 (6.6)	<0.001*	4997 (22.0)A	6363 (23.1)B	3320 (18.5)	293 (6.2)	<0.001
Other	39,725 (23.6)	20,643 (16.7)	8968 (9.1)	1398 (3.2)	<0.001*	2027 (24.1)E	745 (17.6)F	207 (9.3)G	12 (2.3)H	<0.001*	4335 (19.1)A	4025 (14.6)B	1578 (8.8)	193 (4.1)d	<0.001*
Comorbidities (%)															
CHF	10,433 (6.2)	19,567 (15.8)	21,762 (22.0)	11,749 (26.7)	<0.001*	261 (3.1)E	401 (9.5)F	352 (15.9)	113 (21.3)	<0.001*	1365 (6.0)A	3187 (11.5)B	3337 (18.6)C	1313 (27.6)D	<0.001*
COPD	9126 (5.4)	31,602 (25.6)	35,249 (35.6)	16,013 (36.4)	<0.001*	258 (3.1)E	751 (17.7)F	530 (23.9)G	152 (28.6)H	<0.001*	1355 (6.0)a	5023 (18.2)B	4287 (23.8)C	1253 (26.3)D	<0.001*
Alpha 1 antitrypsin deficiency	1934 (1.1)	1919 (1.6)	1584 (1.6)	645 (1.5)	<0.001	388 (4.6)E	204 (4.8)F	93 (4.2)G	18 (3.4)H	0.383	1741 (7.7)A	1872 (6.8)B	1068 (5.9)C	267 (5.6)D	<0.001*
CKD	6249 (3.7)	10,992 (8.9)	12,989 (13.1)	8607 (19.6)	<0.001*	191 (2.3)E	254 (6.0)	224 (10.1)G	93 (17.5)H	<0.001*	1992 (8.8)A	3711 (13.4)B	3349 (18.6)C	1340 (28.2)D	<0.001*
Type II DM	18,853 (11.2)	26,075 (21.1)	22,228 (22.5)	10,010 (22.8)	<0.001*	736 (8.7)E	655 (15.5)F	342 (15.4)	112 (21.1)	<0.001*	3353 (14.7)A	6173 (22.4)	4545 (25.3)C	1490 (31.3)D	<0.001*
Anemia	3335 (2.0)	5101 (4.1)	5305 (5.4)	2822 (6.4)	<0.001*	327 (3.9)E	218 (5.1)F	128 (5.8)G	36 (6.8)H	<0.001*	2507 (11.0)A	3198 (11.6)B	2143 (11.9)C	638 (13.4)D	<0.001*
Elixhauser comorbidity score	18.89 (11.90)	21.83 (12.28)	23.05 (12.33)	23.92 (12.38)	<0.001*	21.54 (11.78)e	24.01 (11.94)	25.26 (12.27)	26.75 (12.06)h	<0.001*	23.11 (12.11)A	25.11 (12.48)	26.35 (12.62)	28.11 (12.68)	<0.001*
Clinical outcomes															
Disposition of patient upon discharge (%)															
Transfer to short‐term hospital	3145 (1.9)	2886 (2.4)	2720 (2.8)	1114 (2.6)	<0.001	206 (2.5)E	123 (3.0)f	44 (2.1)g	12 (2.4)	0.137	840 (3.9)A	792 (3.1)B	531 (3.2)	95 (2.2)d	<0.001
Transfer other: SNF, ICF	12,511 (7.5)	13,763 (11.4)	19,531 (20.3)	13,516 (31.9)	<0.001*	354 (4.3)E	441 (10.8)	432 (20.6)	189 (37.2)h	<0.001*	1553 (7.2)a	3611 (14.0)B	3887 (23.3)C	1476 (33.8)	<0.001*
Transfer to Home Health Care (HHC)	4989 (3.0)	10,033 (8.3)	13,014 (13.5)	8308 (19.6)	<0.001*	245 (3.0)	315 (7.7)	280 (13.3)	91 (17.9)	<0.001*	1868 (8.7)A	3365 (13.0)B	2993 (17.9)C	1062 (24.3)D	<0.001*
Against medical advice (AMA)	11,741 (7.0)	6186 (5.1)	3320 (3.5)	731 (1.7)	<0.001*	583 (7.1)	205 (5.0)	60 (2.9)	12 (2.4)	<0.001*	1258 (5.8)A	1158 (4.5)B	444 (2.7)C	47 (1.1)D	<0.001*
Routine discharge	134,427 (80.6)	88,196 (72.9)	57,642 (59.9)	18,759 (44.2)	<0.001	6848 (83.1)	3014 (73.5)	1284 (61.1)	204 (40.2)	<0.001	15,988 (74.3)A	16,911 (65.5)B	8838 (52.9)C	1693 (38.7)D	<0.001
Total charges in $ (median (IQR))	38,755 [21,686, 76,623]	42,815 [23,899, 82,298]	45,692 [25,439, 86,378]	45,754 [2586, 85,411]	<0.001*	29,551 [17,834, 51,553]E	33,731 [20,089, 60,593]F	38,716 [22,369, 69,338]G	40,277 [24,169, 70,021]H	<0.001*	39,980 [23,658, 71,792]a	41,126 [23,870, 73,411]B	43,803 [25,462, 79,360]C	45,018 [26,083, 80,210]	<0.001*
Length of stay in days (median (IQR))	4.0 [2.0, 7.0]	4.0 [2.0, 7.0]	4.0 [3.0, 8.0]	4.0 [3.0, 8.0]	<0.001	3.0 [2.0, 5.0]	4.0 [2.0, 6.0]	4.0 [2.0, 7.0]	4.0 [3.0, 7.0]	<0.001	4.0 [2.0, 6.0]	4.0 [2.0, 7.0]	4.0 [3.0, 7.0]	4.0 [3.0, 7.0]	<0.001*
In‐hospital mortality (%)	1543 (0.9)	2416 (2.0)	2645 (2.7)	1492 (3.4)	<0.001*	184 (2.2)	133 (3.1)	116 (5.2)g	23 (4.3)H	<0.001*	1230 (5.4)A	1760 (6.4)B	1275 (7.1)C	385 (8.1)D	<0.001*
Number of ICD‐10‐CM on discharge (mean (SD))	12.07 (5.69)	14.89 (6.18)	16.59 (6.32)	17.92 (6.27)	<0.001*	13.92 (5.67)	16.40 (5.90)	17.87 (6.22)	19.58 (6.25)	<0.001*	16.40 (6.00)A	17.52 (6.17)B	18.64 (6.18)C	19.99 (6.09)D	<0.001*
SDOHr (%)	24,223 (14.4)	13,835 (11.2)	6852 (6.9)	1316 (3.0)	<0.001*	422 (5.0)E	257 (6.1)F	115 (5.2)G	17 (3.2)H	0.011	1181 (5.2)A	1729 (6.3)B	727 (4.0)C	90 (1.9)	<0.001*
Sarcopenia	7956 (4.7)	12,073 (9.8)	13,816 (14.0)	6947 (15.8)	<0.001*	1029 (12.2)E	744 (17.6)F	468 (21.1)	128 (24.1)	0.016	3596 (15.8)A	4674 (16.9)B	3611 (20.1)C	1008 (21.2)D	<0.001*
Frailty (%)															
Low risk	114,245 (67.8)	64,209 (52.0)	39,100 (39.5)	12,514 (28.5)	<0.001*	4553 (54.0)E	1775 (41.9)F	749 (33.7)G	119 (22.4)H	<0.001*	11,329 (49.8)A	12,150 (44.0)B	6566 (36.5)C	1294 (27.2)	<0.001*
Intermediate risk	52,485 (31.1)	55,852 (45.2)	54,265 (54.8)	27,390 (62.3)	<0.001	3769 (44.7)E	2342 (55.3)F	1349 (60.8)G	364 (68.5)H	<0.001*	11,016 (48.4)A	14,611 (52.9)B	10,606 (59.0)C	3121 (65.6)D	<0.001*
High risk	1805 (1.1)	3483 (2.8)	5571 (5.6)	4040 (9.2)	<0.001*	104 (1.2)E	117 (2.8)F	122 (5.5)G	48 (9.0)h	<0.001*	405 (1.8)A	849 B(3.1)b	803 (4.5)C	344 (7.2)D	<0.001*
Both sarcopenia and frailty	4756 (2.8)	8443 (6.8)	10,551 (10.7)	5791 (13.2)	<0.001	636 (7.5)E	551 (13.0)F	363 (16.4)G	103 (19.4)H	<0.001*	2408 (10.6)A	3318 (12.0)B	2711 (15.1)C	813 (17.1)D	<0.001

*Note*: “A” comparing <50 AUD versus <50 alcohol‐associated cirrhosis *p* < 0.001. “B” comparing 50–60 AUD versus 50–60 alcohol‐associated cirrhosis *p* < 0.001. “C” comparing 61–70 AUD versus 61–70 alcohol‐associated cirrhosis *p* < 0.001. “D” comparing 71–80 AUD versus 71–80 alcohol‐associated cirrhosis *p* < 0.001. “a” comparing <50 AUD versus <50 alcohol‐associated cirrhosis *p* < 0.05. “b” comparing 50–60 AUD versus 50–60 alcohol‐associated cirrhosis *p* < 0.05. “c” comparing 61–70 AUD versus 61–70 alcohol‐associated cirrhosis *p* < 0.05. “d” comparing 71–80 AUD versus 71–80 alcohol‐associated cirrhosis *p* < 0.05. “E” comparing <50 AUD versus <50 ALD *p* < 0.001. “F” comparing 50–60 AUD versus 50–60 ALD *p* < 0.001. “G” comparing 61–70 AUD versus 61–70 ALD *p* < 0.001. “H” comparing 71–80 AUD versus 71–80 ALD *p* < 0.001. “e” comparing <50 AUD versus <50 alcohol‐associated cirrhosis *p* < 0.05. “f” comparing 50–60 AUD versus 50–60 alcohol‐associated cirrhosis *p* < 0.05. “g” comparing 61–70 AUD versus 61–70 alcohol‐associated cirrhosis *p* < 0.05. “h” comparing 71–80 AUD versus 71–80 alcohol‐associated cirrhosis *p* < 0.05.

Abbreviations: CHF, congestive heart failure; CKD, chronic kidney disease; COPD, chronic obstructive pulmonary disease; ICF, intermediate care facility; SDOHr, social determinants of health risk factor; SNF, skilled nursing facility; Type II DM, type II diabetes mellitus.

* Post hoc analysis of ANOVA with Tukey HSD reveals statistically significant differences among all groups within either GMP or AUD groups.

When we performed multivariate logistic regression across different age groups (Table 7), we determined that AUD + alcohol‐associated cirrhosis had the highest odds for mortality, followed by AUD + ALD and AUD alone. Overall, we also determined that AUD + alcohol‐associated cirrhosis had higher odds for sarcopenia + frailty, frailty, and sarcopenia across all the age groups compared to AUD + ALD and AUD alone. We then performed a sub‐analysis on those with alpha 1 antitrypsin deficiency (Table 8) and found that this population was more likely to have sarcopenia, frailty, or sarcopenia + frailty whether or not they had AUD.

**TABLE 7:** Regression analyses comparing cohorts across age groups.

	AUD vs. GMP < 50 years		AUD + ALD vs. GMP < 50 years		AUD + alcohol‐associated cirrhosis vs. GMP < 50 years	
	Unadj OR (95% CI)	Adj OR (95% CI)	Unadj OR (95% CI)	Adj OR (95% CI)	Unadj OR (95% CI)	Adj OR (95% CI)
Sarcopenia	**1.09 (1.05–1.14)**	**1.23 (1.18–1.28)**	**3.06 (2.84–3.30)**	**3.16 (2.92–3.41)**	**4.13 (3.93–4.34)**	**4.20 (3.98–4.42)**
Frailty	**1.23 (1.20–1.25)**	**1.35 (1.32–1.38)**	**2.19 (2.10–2.30)**	**2.27 (2.17–2.38)**	**2.60 (2.52–2.68)**	**2.60 (2.52–2.68)**
Sarcopenia + frailty	**1.09 (1.03–1.15)**	**1.28 (1.21–1.36)**	**3.07 (2.80–3.37)**	**3.25 (2.95–3.57)**	**4.45 (4.18–4.74)**	**4.55 (4.27–4.86)**
CoH	**0.89 (0.89–0.90)**	**0.91 (0.90–0.92)**	**1.03 (1.01–1.06)**	**1.04 (1.01–1.06)**	**1.41 (1.39–1.43)**	**1.36 (1.34–1.38)**
LoS	**1.13 (1.11–1.14)**	**1.16 (1.14–1.18)**	**1.23 (1.19–1.28)**	**1.24 (1.20–1.29)**	**1.40 (1.36–1.43)**	**1.39 (1.36–1.43)**
In‐hospital mortality	0.95 (0.87–1.04)	1.01 (0.92–1.11)	**2.30 (1.94–2.70)**	**2.26 (1.91–2.67)**	**5.88 (5.35–6.46)**	**5.50 (5.00–6.07)**
Discharge to home	**0.95 (0.93–0.97)**	**0.94 (0.92–0.96)**	1.04 (0.98–1.10)	**1.07 (1.01–1.14)**	**0.57 (0.55–0.59)**	**0.59 (0.57–0.61)**

	AUD vs. GMP 50–60 years		AUD + ALD vs. GMP 50–60 years		AUD + alcohol‐associated cirrhosis vs. GMP 50–60 years	
	Unadj OR (95% CI)	Adj OR (95% CI)	Unadj OR (95% CI)	Adj OR (95% CI)	Unadj OR (95% CI)	Adj OR (95% CI)
Sarcopenia	**1.28 (1.23–1.33)**	**1.46 (1.40–1.52)**	**2.52 (2.31–2.75)**	**2.67 (2.44–2.91)**	**2.41 (2.30–2.52)**	**2.60 (2.48–2.73)**
Frailty	**1.14 (1.12–1.17)**	**1.30 (1.27–1.33)**	**1.71 (1.60–1.82)**	**1.81 (1.70–1.94)**	**1.57 (1.52–1.62)**	**1.66 (1.61–1.71)**
Sarcopenia + frailty	**1.31 (1.25–1.37)**	**1.53 (1.46–1.61)**	**2.67 (2.42–2.95)**	**2.86 (2.59–3.16)**	**2.44 (2.31–2.58)**	**2.66 (2.51–2.81)**
CoH	**0.91 (0.90–0.92)**	**0.94 (0.93–0.95)**	**0.95 (0.92–0.98)**	**0.96 (0.94–0.99)**	**1.14 (1.13–1.16)**	**1.12 (1.11–1.14)**
LoS	**1.03 (1.01–1.04)**	**1.07 (1.05–1.09)**	**1.17 (1.12–1.22)**	**1.19 (1.14–1.25)**	**1.21 (1.19–1.24)**	**1.23 (1.20–1.26)**
In‐hospital mortality	0.95 (0.88–1.03)	1.05 (0.97–1.13)	**1.54 (1.28–1.85)**	**1.57 (1.29–1.89)**	**3.24 (2.99–3.52)**	**3.23 (2.97–3.51)**
Discharge to home	**1.13 (1.10–1.16)**	**1.06 (1.04–1.09)**	**1.12 (1.04–1.20)**	**1.11 (1.03–1.19)**	**0.72 (0.69–0.74)**	**0.70 (0.68–0.73)**

	AUD vs. GMP 61–70 years		AUD + ALD vs. GMP 61–70 years		AUD + alcohol‐associated cirrhosis vs. GMP 61–70 years	
	Unadj OR (95% CI)	Adj OR (95% CI)	Unadj OR (95% CI)	Adj OR (95% CI)	Unadj OR (95% CI)	Adj OR (95% CI)
Sarcopenia	**1.51 (1.46–1.56)**	**1.72 (1.66–1.78)**	**2.48 (2.23–2.76)**	**2.72 (2.44–3.03)**	**2.34 (2.23–2.45)**	**2.56 (2.44–2.69)**
Frailty	**1.30 (1.27–1.33)**	**1.51 (1.47–1.54)**	**1.67 (1.53–1.82)**	**1.82 (1.66–2.00)**	**1.48 (1.43–1.53)**	**1.59 (1.54–1.65)**
Sarcopenia + frailty	**1.53 (1.48–1.59)**	**1.78 (1.71–1.85)**	**2.51 (2.23–2.82)**	**2.75 (2.44–3.10)**	**2.28 (2.17–2.40)**	**2.48 (2.35–2.61)**
CoH	1.00 (0.99–1.01)	**1.03 (1.02–1.04)**	1.01 (0.97–1.05)	1.03 (0.99–1.07)	**1.15 (1.13–1.16)**	**1.14 (1.12–1.15)**
LoS	**1.05 (1.03–1.06)**	**1.09 (1.08–1.11)**	**1.21 (1.14–1.28)**	**1.23 (1.17–1.31)**	**1.21 (1.18–1.24)**	**1.23 (1.17–1.31)**
In‐hospital mortality	**0.89 (0.83–0.94)**	0.96 (0.90–1.02)	**1.78 (1.46–2.15)**	**1.88 (1.54–2.28)**	**2.46 (2.28–2.65)**	**2.50 (2.31–2.69)**
Discharge to home	**1.03 (1.01–1.06)**	**0.94 (0.92–0.96)**	1.02 (0.93–1.11)	0.96 (0.88–1.05)	**0.72 (0.69–0.74)**	**0.68 (0.66–0.70)**

	AUD vs. GMP 71–80 years		AUD + ALD vs. GMP 71–80 years		AUD + alcohol‐associated cirrhosis vs. GMP 71–80 years	
	Unadj OR (95% CI)	Adj OR (95% CI)	Unadj OR (95% CI)	Adj OR (95% CI)	Unadj OR (95% CI)	Adj OR (95% CI)
Sarcopenia	**1.48 (1.43–1.53)**	**1.68 (1.61–1.74)**	**2.50 (2.04–3.05)**	**2.70 (2.19–3.30)**	**2.12 (1.97–2.28)**	**2.21 (2.05–2.39)**
Frailty	**1.43 (1.39–1.47)**	**1.63 (1.58–1.68)**	**1.97 (1.61–2.43)**	**2.09 (1.71–2.59)**	**1.52 (1.43–1.63)**	**1.61 (1.51–1.72)**
Sarcopenia + frailty	**1.53 (1.47–1.59)**	**1.76 (1.69–1.84)**	**2.43 (1.94–3.00)**	**2.63 (2.10–3.27)**	**2.08 (1.92–2.25)**	**2.19 (2.02–2.38)**
CoH	**1.07 (1.06–1.08)**	**1.11 (1.09–1.12)**	1.06 (0.98–1.15)	**1.09 (1.01–1.17)**	**1.17 (1.14–1.20)**	**1.14 (1.11–1.17)**
LoS	**1.06 (1.05–1.08)**	**1.11 (1.09–1.13)**	**1.25 (1.12–1.40)**	**1.28 (1.15–1.43)**	**1.22 (1.17–1.26)**	**1.23 (1.18–1.28)**
In‐hospital mortality	**0.81 (0.76–0.87)**	**0.84 (0.79–0.91)**	1.05 (0.67–1.56)	1.02 (0.64–1.53)	**2.04 (1.82–2.28)**	**1.91 (1.70–2.14)**
Discharge to home	**0.92 (0.90–0.94)**	**0.82 (0.79–0.84)**	**0.77 (0.65–0.92)**	**0.72 (0.60–0.86)**	**0.68 (0.64–0.73)**	**0.62 (0.59–0.66)**

*Note*: CoH and LoS represent log‐transformed linear regression models. Mortality and discharge to home represent logistic regression. Model adjusted for race, sex, and comorbidities as determined by Elixhauser comorbidity score. Bolded odds ratios are statistically significant.

**TABLE 8:** Association of alpha 1 antitrypsin deficiency (AAD) with sarcopenia/frailty/sarcopenia + frailty.

	No AUD			AUD		
	No AAD	AAD	*p*‐Value	No AAD	AAD	*p*‐Value
*n*	285,176	3915		523,648	11,921	
Age (mean (SD))	62.07 (18.85)	62.56 (16.41)	0.107	53.69 (14.53)	54.55 (12.57)	<0.001
Female (%)	147,524 (51.7)	1943 (49.6)	0.009	147,456 (28.2)	3738 (31.4)	<0.001
Race (%)			0.001			<0.001
White	183,626 (65.7)	2401 (62.7)		326,643 (63.6)	7306 (62.6)	
Black	46,768 (16.7)	708 (18.5)		104,724 (20.4)	1951 (16.7)	
Other	49,185 (17.6)	718 (18.8)		81,852 (15.9)	2419 (20.7)	
Mortality	8342 (2.9)	240 (6.1)	<0.001	13,029 (2.5)	695 (5.8)	<0.001
Sarcopenia (%)	25,619 (9.0)	873 (22.3)	<0.001	55,351 (10.6)	2741 (23.0)	<0.001
Frailty (%)			<0.001			<0.001
Low risk	139,920 (49.1)	1040 (26.6)		267,251 (51.0)	3694 (31.0)	
Intermediate risk	130,747 (45.8)	2506 (64.0)		237,661 (45.4)	7507 (63.0)	
High risk	14,509 (5.1)	369 (9.4)		18,736 (3.6)	720 (6.0)	
Sarcopenia + frailty (%)	19,392 (6.8)	732 (18.7)	<0.001	40,146 (7.7)	2106 (17.7)	<0.001
COPD	55,804 (19.6)	790 (20.2)	0.349	106,993 (20.4)	2148 (18.0)	<0.001

*Note*: *p*‐Value represents *t*‐test for quantitative variables and *z*‐test of proportions for categorical variables.

## DISCUSSION

The incidence and consequences of alcohol overuse in the elderly population are currently understudied. In our analysis, we utilized the NIS database to investigate the impact of alcohol overuse on clinical outcomes across the lifespan.

Our analyses of the prevalence and outcomes of alcohol overuse in the elderly in a large national database of hospitalized patients in the United States found that patients with AUD overall had higher rates of sarcopenia, frailty, healthcare costs, length of stay, and discharge status. These higher rates of adverse clinical outcomes were noted across all age groups. Mortality rates were higher in the AUD group compared to the GMP group across all age groups except for those >70. However, this may be due to survivor bias, possibly due to selective survival of more resilient individuals or survival of those with less severe alcohol‐related organ injury. Diagnostic under‐recognition of AUD in older adults is also common (Joshi et al., 2021), which may have contributed to the survivor bias. When examining comorbidities, patients with AUD had lower rates of CHF, CKD, and anemia as compared to the GMP, but had higher rates of COPD and alpha 1 antitrypsin deficiency across all age groups. This may be explained by differing admission etiologies, where AUD admissions may be precipitated by alcohol‐related issues such as withdrawal or intoxication, whereas GMP admissions occur due to decompensated chronic diseases like CHF, CKD, or anemia. In terms of higher rates of COPD in AUD patients, it has been demonstrated previously that patients who drink alcohol are more likely to smoke cigarettes as a graded effect (Garnett et al., 2022). Concurrent alcohol abuse is also common in COPD and associated with adverse outcomes (MacMurdo et al., 2021). AATD are particularly prone to adverse outcomes from smoking and alcohol intake given they are predisposed to both lung and liver disease; this is due to the toxic loss of function of AAT in the lung (causing emphysema) and the toxic gain of function of AAT in the liver (misfolded protein causing liver injury) (Attaway et al., 2019; Stoller, 2024). We also found that alpha 1 antitrypsin deficiency was common in those with AUD + alcohol‐associated cirrhosis and AUD + ALD. Whether or not AATD should be screened in patients with AUD is an area of future investigation. Finally, a sub‐analysis determined that alpha‐1 antitrypsin deficiency was more commonly associated with sarcopenia, frailty, or sarcopenia + frailty. This is consistent with previous studies analyzing alpha‐1 antitrypsin deficiency in COPD patients (Duckers et al., 2010), although the mechanisms underlying sarcopenia in alpha‐1 antitrypsin deficiency remain to be elucidated.

We then examined patients with sarcopenia and patients with frailty and those with and without AUD across all age groups. In‐hospital mortality was higher among those with AUD and sarcopenia and frailty, as compared to those without AUD. Our regression analysis also demonstrated that there is an independent effect of AUD associated with adverse clinical outcomes. Sarcopenia is the age‐associated progressive loss of skeletal muscle mass and function (Cruz‐Jentoft & Sayer, 2019; Fielding et al., 2011), and in this analysis, we utilized the billing code of muscle loss phenotype (Attaway et al., 2024) as a marker of sarcopenia. Prior studies have found that sarcopenia and frailty are associated with and are strong predictors of worse clinical outcomes including higher rates of mortality, longer hospital stays, higher healthcare costs, and increased rates of hospital readmission (Antunes et al., 2017; Bernabeu‐Wittel et al., 2019; Gariballa & Alessa, 2013; Xu et al., 2022). Independently, AUD has been found to be associated with significantly increased mortality rates (Pell & D'alonzo, 1973; Roerecke & Rehm, 2013; Westman et al., 2015).

Interestingly, alcohol consumption has not been found to be directly associated with sarcopenia (Bu et al., 2024; Skinner et al., 2023), although these studies were performed in population‐level analyses like the UK Biobank and not in completely hospitalized cohorts such as the patients in our study. There is overall limited literature studying the effect of AUD in hospitalized patients with sarcopenia and the effects on overall health outcomes. In our cohort, we noted that mortality was not greater in those with AUD alone versus GMP but that sarcopenia and frailty were significantly increased across all age cohorts. This suggests that the negative aspects of AUD may impact muscle loss prior to the development of alcohol‐associated liver disorders like cirrhosis or ALD. There are a number of ongoing studies suggesting that muscle breakdown due to alcohol releases amino acids like glutamine that can overload the liver with nitrogen and cause worsening liver stress (Dasarathy et al., 2017). There are also a number of studies that demonstrate how skeletal muscle may help clear ammonia, and the lack of ammonia clearance can worsen hepatic encephalopathy (Jindal & Jagdish, 2019). Finally, sarcopenia has been known to increase insulin resistance, which can drive non‐alcohol‐associated fatty liver disease and its progression to steatohepatitis (Li et al., 2019). Given these findings, our results highlight how sarcopenia and frailty can occur early in those with AUD and may even contribute to and compound the effects of alcohol‐associated liver diseases.

When conducting a sub‐analysis on patients with different etiologies of liver disease, we found that sarcopenia and frailty rates were higher in patients with alcohol‐associated liver disease (ALD) and alcohol‐associated cirrhosis as compared to patients with AUD across all age ranges. Prior literature has shown that patients with cirrhosis are at higher risk of sarcopenia, significantly increasing the risk of liver‐related mortality (Dhaliwal & Armstrong, 2020; Ebadi et al., 2019). Additionally, in‐hospital mortality was higher in patients with both ALD and cirrhosis as compared to patients with AUD across all age groups, suggesting that the severity of liver disease and age both contribute to the severity of outcomes.

Prior literature has found that alcohol contributes to variations in neurobehavioral effects in the elderly including cognitive impairment, but to our knowledge, this is the first study examining the trends across ages of various outcomes in patients with alcohol overuse (Deak & Savage, 2019). Although our dataset consists of a large, unbiased cohort of hospitalized patients and their associated outcomes, we recognize that there are limitations to our study. Prior studies have found that there may be errors in coding data, and there is not always consistency between a patient's medical record diagnosis and billing codes used by physicians (Burns et al., 2011). Billing codes are also not always accurate due to mistakes during the entry of the code.

In conclusion, using a large national dataset, we show that there is a significant association between AUD on adverse clinical outcomes (sarcopenia, frailty, and healthcare utilization) with increasing age. Furthermore, a diagnosis of sarcopenia and frailty confers a greater mortality risk than either alone in patients with AUD with increasing age. Our findings highlight the need for targeted alcohol interventions such as frailty and sarcopenia screening in older hospitalized patients with AUD or the integration of addiction medicine within geriatric care.

## FUNDING INFORMATION

This work was supported by National Institutes of Health (1K08HL168348) (AA).

## CONFLICT OF INTEREST STATEMENT

All authors state that they have no conflict of interest.

## Supporting information


Table S1

## Data Availability

The data used in this study were obtained from the Healthcare Cost and Utilization Project (HCUP) Nationwide Inpatient Sample (NIS), which is publicly available from the HCUP Central Distributor (https://www.hcup‐us.ahrq.gov/). Access to the data requires a data use agreement and approval from the HCUP Central Distributor. The authors do not have permission to share the dataset directly.
