# Supplementary material for: Age‐related impact of outcomes in hospitalized patients with alcohol overuse
Source: Alcohol Clin Exp Res (Hoboken). 2025 Dec 1;50(1):e70219. doi: 10.1111/acer.70219 (PMC12828099; doi:10.1111/acer.70219)
Supplement: Supplementary file 1 — Table S1 [file ACER-50-0-s001.docx]

**Supplementary Table 1 - Disease definitions**

| **Diagnosis** | **ICD codes** |
| --- | --- |
| Alcohol use disorder | F10.0*, F10.1, F10.2, F10.9x^1^ excluding “in remission” specifiers (F10.11, F10.21, F10.91) |
| AUD + Alcoholic liver disease | F10.0*, F10.1, F10.2, F10.9x^1^ excluding “in remission” specifiers (F10.11, F10.21, F10.91)  AND  K70.0*, K70.1, K70.2*, K70.9 |
| AUD + Alcoholic cirrhosis | F10.0*, F10.1, F10.2, F10.9x^1^ excluding “in remission” specifiers (F10.11, F10.21, F10.91)  AND  K70.3 |
| Alpha 1 antitrypsin deficiency | E88.01 |
| Social determinants of health Z-codes | Z55-57, Z59, Z60, Z62-65, Z75 |
| *Codes that were not present in the database. ^1^10.90 was not present in the database, but codes with a fourth digit were present (i.e 10.92-10.99). | |
